# Iron Oxide Nanoparticles Stimulates Extra-Cellular Matrix Production in Cellular Spheroids

**DOI:** 10.3390/bioengineering4010004

**Published:** 2017-01-21

**Authors:** Megan Casco, Timothy Olsen, Austin Herbst, Grace Evans, Taylor Rothermel, Lauren Pruett, Dan Simionescu, Richard Visconti, Frank Alexis

**Affiliations:** 1 Department of Bioengineering, Clemson University, 301 Rhodes Research Center, Clemson, SC 29634, USA; mcasco@g.clemson.edu (M.C.); trolsen@clemson.edu (T.O.); aherbst@clemson.edu (A.H.); gracee@g.clemson.edu (G.E.); trother@g.clemson.edu (T.R.); lpruett@g.clemson.edu (L.P.); 2 Department of Regenerative Medicine and Cell Biology, Medical University of South Carolina 173 Ashley Avenue—BSB 601, Charleston, SC 29425, USA; 3 Institute of Biological Interfaces of Engineering, Department of Bioengineering, Clemson University, 401-2 Rhodes Engineering Research Center, Clemson, SC 29634, USA

**Keywords:** tissue engineering, spheroids, magnetic nanoparticles, extracellular matrix

## Abstract

Nanotechnologies have been integrated into drug delivery, and non-invasive imaging applications, into nanostructured scaffolds for the manipulation of cells. The objective of this work was to determine how the physico-chemical properties of magnetic nanoparticles (MNPs) and their spatial distribution into cellular spheroids stimulated cells to produce an extracellular matrix (ECM). The MNP concentration (0.03 mg/mL, 0.1 mg/mL and 0.3 mg/mL), type (magnetoferritin), shape (nanorod—85 nm × 425 nm) and incorporation method were studied to determine each of their effects on the specific stimulation of four ECM proteins (collagen I, collagen IV, elastin and fibronectin) in primary rat aortic smooth muscle cell. Results demonstrated that as MNP concentration increased there was up to a 6.32-fold increase in collagen production over no MNP samples. Semi-quantitative Immunohistochemistry (IHC) results demonstrated that MNP type had the greatest influence on elastin production with a 56.28% positive area stain compared to controls and MNP shape favored elastin stimulation with a 50.19% positive area stain. Finally, there are no adverse effects of MNPs on cellular contractile ability. This study provides insight on the stimulation of ECM production in cells and tissues, which is important because it plays a critical role in regulating cellular functions.

## 1. Introduction

Nanotechnologies have been integrated into non-invasive imaging and drug and gene delivery applications, nanostructured scaffolds, and for the manipulation of cells [1,2]. Magnetic nanoparticles (MNPs) have been used in cellular spheroids for the alignment and patterning of tissues mediated by magnetic forces [1,2,3,4,5,6,7,8,9]. This technique is most effective in the manipulation of samples from a distance, for the precise placement and manipulation of samples, and for the control of both the exposure time and frequency [2,6]. For example, in one such study binding the magnetic nanoparticles to the surface of cells, Dobson applied an external magnetic force to remotely stimulate and control cell function by precisely targeting and activating specific ion channels or cell surface receptors on these cells [10]. Magnetic forces are also useful for facilitating the seeding of magnetic cells deeper into scaffolds for various tissue engineering applications, resulting in a higher scaffold-seeding efficiency than traditional seeding methods [1,8].

Though the method of incorporating MNPs within cells and tissues varies, it nonetheless guides the biological response. One common method of incorporation entails the cellular internalization of the MNPs, which is the incubation of cells with MNPs to facilitate the uptake into the cells under the influence of magnetic fields [2,6,11,12]. Another method entails the dispersion of MNPs within the extracellular matrix (ECM). Although the MNPs are mostly located outside the cells, they are homogenously dispersed throughout the ECM. Unfortunately both methods adversely affect the viability, activity, and phenotypic stability of the cells [6,13]. Our novel procedure, however, known as the Janus method for the fabrication of magnetic cellular spheroids creates two distinct domains: the extracellular MNP and the cells [6,11,12]. Unlike the internalization and dispersal methods, our method is unique in that it is characterized by a limited interaction between cells and MNPs that increases cell viability over time and thus limits the adverse effects on the cell phenotype.

As the biological response to MNPs is related to the nano-bio interface, it may affect the viability, phenotype, cellular mechanisms, and cellular functions [13]. It is now known that the chemical composition, surface functionalization, and shape of nanoparticles can affect the interactions of the nanoparticle with the surrounding environment [13,14]. The nano-bio interface is composed of the interactions between the surface of the nanomaterial and the surface of the biological components (i.e., proteins and membranes) [13]. The chemical composition of a nanoparticle can determine which ions, proteins, or other organic materials in the medium will adhere to the surface of the nanoparticle [14]. A nanoparticle’s shape directly influences the uptake of that nanoparticle into the cell by determining how the nanoparticle will interact with the cell membrane [14,15]. However, it is important to consider the nano-bio interface to determine if the nanoparticles affect the stimulation of cells which is necessary to produce ECM.

ECM is a dynamic, highly organized structure that regulates cellular functions such as proliferation and differentiation [16,17,18,19,20]. Collagen, types I and IV, elastin and fibronectin are important proteins of ECM. The role of collagen is to provide strength and structural integrity of tissues [18,19,20]. Fibronectin is important for the binding of cells to the ECM and elastin provides flexibility [17,18,20]. Iron is known to increase both collagen and elastin synthesis without adversely affecting other proteins [21,22,23]. It is also an important physiological ion that is essential for cellular functions including redox activity and cell proliferation [21,23,24].

Iron is not the only element that has an effect on ECM production. Indeed, the effects of silver and gold have also been researched. Silver nanoparticles are commonly used in dermal applications because of their positive influence on collagen production and for the promotion of the semi-alignment of collagen fibers [25,26]. Although gold nanoparticles inhibit advanced glycation end products (AGE), they can cause abnormal ECM constructs that can lead to a decrease in proliferation, adhesion, and motility, thus making them ill-suited for anti-aging purposes [27]. Therefore, for the purposes of this paper the focus is on iron oxide nanoparticles.

In this work we assess the ability of different MNP formulations to stimulate cells to produce ECM for purposes of determining how the physico-chemical properties of MNPs and their spatial distribution into spheroids can stimulate cells to produce ECM. The hypothesis is that the shape and surface properties of iron based nanoparticles can stimulate cells to produce specific types of ECM proteins in response to alterations of nano-bio interface. The rationale is that if the influence of iron oxide (IO) concentration, shape, and type, and method of incorporation on ECM production can be understood, then it will be possible to stimulate cells to produce specific ECM in vitro. Our results demonstrate that by varying IO concentration, incorporating different types and shapes of MNPs, as well as using different incorporation methods stimulation of specific ECM proteins can be achieved. Results also showed no adverse effects on cellular functions of contraction and relaxation due to MNPs.

## 2. Materials and Methods

### 2.1. Cell Culture

Primary rat aortic smooth muscle cells were isolated from the aorta via careful removal of the adventitia and endothelial cell lining, followed by a one hour incubation with trypsin (0.25%) and collagenase (5 units/mL). The cell phenotype was confirmed by staining for α-SMA and SM22-Alpha, phenotypic markers for smooth muscle cells, with only passage numbers under ten used. All cells were maintained in monolayer cultures at 37 °C and 5% of CO_2_ prior to spheroid assembly. SMCs were cultured using Dulbeco’s Modified Eagle Medium:F-12 (ATCC, Manassas, VA, USA, 1:1, DMEM:F-12) supplemented with 10% fetal bovine serum (Atlanta Biologics, Flowery Branch, GA, USA) and 1% penicillin-streptomycin-amphotericin (MediaTech, Inc., Tewksbury, MA, USA).

### 2.2. Spheroid Assembly

Spheroids of six different formulations were prepared using either the Janus method that was developed in our lab or the dispersion method. The Janus method, which is a technique described in our previous publications, is used to incorporate MNPs into spheroids that reduces the interaction between cells and the MNPs by creating two distinct domains of each [2,5].The following five formulations described were prepared using the Janus method: (1) 0.03 mg/mL iron oxide (IO) incorporated into spheroids; (2) 0.1 mg/mL IO incorporated into spheroids; (3) 0.3 mg/mL IO incorporated into spheroids; (4) spheroids formulated with 0.3 mg/mL nanorods; (5) spheroids formulated with 0.3 mg/mL magnetoferritin. The sixth formulation is the incorporation of 0.3 mg/mL IO into spheroids using the dispersion method. To determine the effect of MNP concentration on ECM production, we incorporated 0.03 mg/mL, 0.1 mg/mL, and 0.3 mg/mL IO (Fe_3_O_4_, 20–30 nm, SkySpring Nanomaterials, Inc., Houston, TX, USA) into cellular spheroids. The dispersion method (0.3 mg/mL IO) was used to determine the effect of incorporation method of MNPs on ECM production compared to the Janus method. Non-iron oxide (NIO) spheroids were used as a control for all experiments. Magnetoferritin was then prepared using our previously developed procedure to determine the effect of the type of MNP incorporated into the spheroids [2]. Lastly, Fe_2_O_3_ (0.3 mg/mL IO), 85 nm × 425 nm, NanoArmor nanorods were used to determine the effect of the shape of MNP on ECM production. Equal volumes of solutions containing suspended nanoparticles, collagen (Bovine, Type I, Life Technologies, Carlsbad, CA, USA), and cells in cell culture media were combined and dispensed as hanging drops (15 μL) to form spheroids. Collagen Type I was prepared according to the manufacturer’s recommendations, kept on ice prior to use for all samples, and only added at the time of spheroid fabrication and not continuously to the media. Unless otherwise noted, all spheroids were incorporated into the samples after three days of incubation. Supplementary Figure S2 shows the TEM images of spherical nanoparticles, nanorods, and magnetoferritin.

### 2.3. “Mini” Tissue Processing

The hanging drop method was next used to form Janus magnetic cellular spheroids over a three day period [16]. A single cylindrical magnet (2.5 mm diameter, 5 mm length, pull fore = 1.8 lbs., SuperMagnet Man, Pelham, NY, USA) was attached to the outside bottom of a well on a non-tissue culture treated 12 well plate. 1.0 mL of media was added to the well and 25 spheroids were arranged along the magnet. The spheroids were fused for 48 h and the magnets were then removed. These “mini” tissues were allowed to continue to fuse and contract for 72 h prior to use.

### 2.4. Collagen Quantification

A hydroxyproline assay was next used to quantify the collagen content in the spheroids of the six fabrications. First, spheroids were frozen and lyophilized, then hydrolyzed in 4 N sodium hydroxide at 120 °C for 2 h. Samples were neutralized with 1.4 N citric acid and pH balanced to a range of 7.2 to 7.6. A hydroxyproline standard curve was prepared using known concentrations of collagen for calibration of unknown samples. An aliquot of 200 µL was taken from each sample and incubated with a Chloramine T solution for 15 min at room temperature followed by immersion in a *p*-Dimethylaminobenzaldehyde solution for 15 min at 65 °C. The experimental samples were then prepared as triplicates for optical readings at 550 nm. The unknown samples were calculated based on the hydroxyproline standard curve. Long-term spheroid collagen content studies were normalized to the collagen content of each respective group on Day 3.

### 2.5. Functional Assay

Potassium chloride (KCl) was used to test the functionality of the spheroids and “mini” tissues fabricated by stimulating the smooth muscle cells to contract. Individual spheroids or “mini” tissues were placed into 12 well plates with media and imaged at initial time point. For the control samples the media was replaced with fresh media; for the experimental samples the media was replaced with KCl media (80 mM KCl). After 10 min (30 min for “mini” tissues) the KCl media was replaced with plain media and samples were imaged. The samples were placed in the incubator (37 °C, 5% CO_2_) until they were again imaged at 24, 48, and 72 h intervals with an AMG Evos fl microscope (Fisher Scientific, Hampton, NY, USA). The diameter of the individual spheroids or length of the “mini” tissues were measured from the images taken at each time point. All measurements were compared to initial time points to determine what effect the KCl had on the contractile function of smooth muscle cells.

### 2.6. Histological Examination

All samples were processed and sectioned via paraffin sectioning techniques developed in our lab [28]. The spheroids were then fixed, processed, and stained using hemotoxylin and eosin (H & E), or Masson’s Trichrome. Next, samples were stained via immunohistochemistry at Days 3 and 40 for Collagen Type I and IV, elastin, and fibronectin. The following antibodies were then used for immunolabeling: anti-collagen I antibody (1:200; abcam; ab34710), anti-collagen IV antibody (1:200; abcam; ab6586), anti-fibronectin (1:200; BD Biosciences, Franklin Lakes, NJ, USA; 610077), and anti-elastin (1:50; abcam; ab23748). The prepared slides were analyzed using a BOND-MAX automated Immunohistochemistry (IHC) machine (Leica, Wetzlar, Germany). An automated Novocastra Bond Polymer Refine Detection system (Leica; DS9800) was used to stain and detect protein markers in the tissue samples. Slides were imaged using a Nikon AZ100 multizoom microscope (Nikon Instruments Inc., Mellville, NY, USA). MNP spheroids were compared to NIO samples to analyze the effect of MNP concentration, type and shape and incorporation method by analyzing cellular matrix production. Negative controls for each IHC stain (only counterstained for nuclei) were used as a visual representation to show that the IHC was successful. No calculations, comparisons or normalizations were performed with the negative controls. All quantitative results were obtained by calculating percent area of positive stain using ImageJ and then comparing to the NIO control. Three samples of each formulation were analyzed to confirm a consistency of results.

### 2.7. Statistical Analysis

An Analysis of Variance (ANOVA) was used to statistically determine the presence of any differences amongst the treatment groups. For any differences determined from the ANOVA, a post hoc two tailed T-test was used to determine any significant differences between both treatments tested. The error bars on graphs represent the standard deviation from the mean. All measurements were normalized to the zero time point to remove variance and the same spheroid was measured at the same time point and compared with their respective time zero size.

## 3. Results

**Figure 1:**
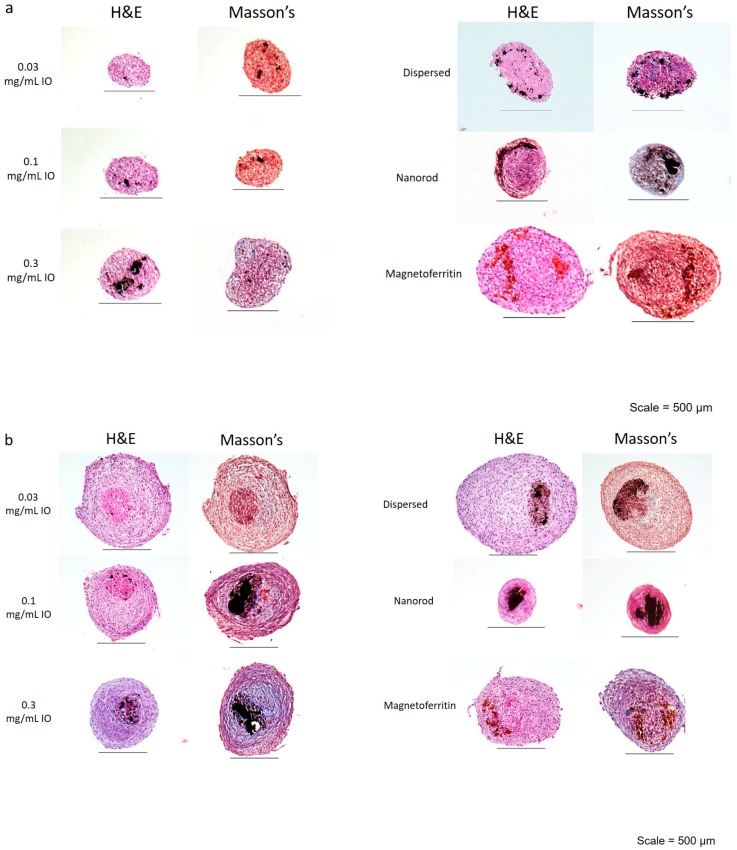
H & E and Masson’s Trichrome Staining for magnetic nanoparticles (MNP) spheroids. Spheroids were fabricated with varying MNP concentrations, type, shape, incorporation method, or no MNP. After (**a**) Day 3 and (**b**) Day 40 time points, spheroids were collected, fixed, and processed for histological examination. The H & E stain demonstrates the presence of nuclei throughout all formulations of spheroids after 3 and 40 days. The Masson’s Trichrome stain demonstrates an increased presence of collagen (represented by the blue in the samples) in the Janus, 0.1 mg/mL iron oxide (IO), and magnetoferritin spheroids on Day 40.

Collagen is an important structural component of tissues and its production within the spheroids is affected by the metallic nanoparticles [29]. Therefore, to visualize nuclei and collagen, the spheroids were histologically sectioned and stained with hemotoxylin and eosin (H & E) and Masson’s Trichrome stains at the time points of days 3 and 40 (Figure 1a,b). Although collagen is the only ECM protein that can be visualized using these methods, neither can specify the presence of a specific type of collagen. Although a histological examination confirmed that over time collagen production increased for all spheroid types, the spheroids with 0.3 mg/mL IO visually exhibited the greatest increase in collagen content. Overall, these results demonstrate that the exposure of these cells to iron oxide increased the production of collagen compared to the NIO control.

**Figure 2:**
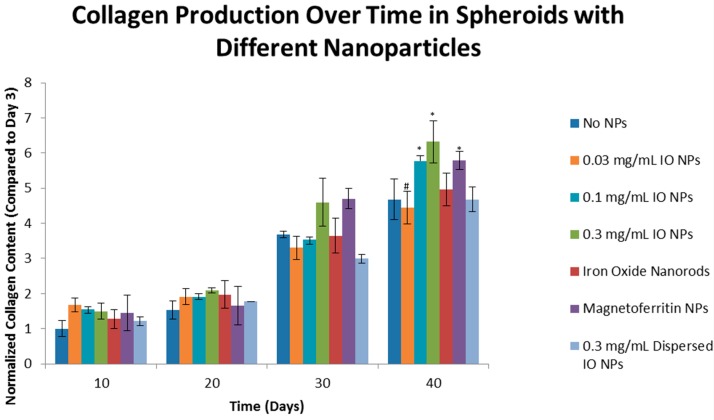
Effect of Concentration, Type, Shape, and Incorporation Method of magnetic nanoparticles on Collagen Synthesis over Time. Results of hydroxyproline assay quantitatively demonstrates that there is an increase in collagen production in all spheroid types over 40 days. When compared to the Day 40 control, no iron oxide (NIO), there is only a statistical difference (*p* < 0.05, as indicated by “*”) for 0.1 mg/mL IO, magnetoferritin, and Janus spheroids. The results indicate that the higher concentrations of iron oxide and biological MNPs produce higher amounts of collagen, relative to the control. The results show that the when the IO formulations are compared to each other, there was a significant difference between Janus, as the control, and 0.03 mg/mL IO (*p* < 0.05, as indicated by “#”). This supports the earlier results that the higher concentrations of IO increase collagen production.

The results of the hydroxyproline assay quantitatively demonstrated an increase of collagen over time for all formulations of spheroids (Figure 2). Regarding the effects of MNP concentration on collagen production, the results further demonstrated that the lower concentration of IO, 0.03 mg/mL IO, did not significantly increase the production of collagen when compared to 0.3 mg/mL IO Day 40 (*p* < 0.05, indicated by “#”). Both 0.1 and 0.3 mg/mL IO, however, did show a significant increase. In summary, the results demonstrated that the incorporation of IO into cellular spheroids caused a significant increase in collagen production when compared to their NIO counterparts at Day 40 (*p* < 0.05, indicated by “*”). Compared to Day 3, Day 40 NIO spheroids had a 4.68 fold greater collagen content, 0.1 mg/mL IO had a 5.78 fold greater collagen content, and 0.3 mg/mL IO had a 6.32 fold greater collagen content. Here we demonstrated the significant effects on how the variance in IO concentrations had on collagen production, which led to the investigation of the nano-bio interface.

To determine the effect of MNP shape on collagen production, nanorod (0.3 mg/mL IO nanorods) MNPs were compared to spherical MNPs. Magnetoferritin was used to determine the effect of a biological corona composed of an apoferritin shell on the production of collagen in cellular spheroids. The dispersion method of incorporating MNPs into spheroids was used to determine the effect of the incorporation. The results from the hydroxyproline assay demonstrated that the nanorod-shaped MNP had no significant effect on collagen production in cellular spheroids. Results also indicated a significant increase of collagen production with the use of our Janus method compared to the results of the dispersed method. Therefore, the method of incorporation does affect collagen production. Furthermore, the results demonstrated that magnetoferritin significantly increased collagen production when compared to the NIO control. Compared to the results on Day 3, Day 40 NIO spheroids exhibited a 4.68 fold greater increase in collagen content and magnetoferritin had a 5.79 fold greater collagen content. We have demonstrated that the concentration and type of MNP and the method used to incorporate MNPs affects the level of collagen production over time.

**Figure 3:**
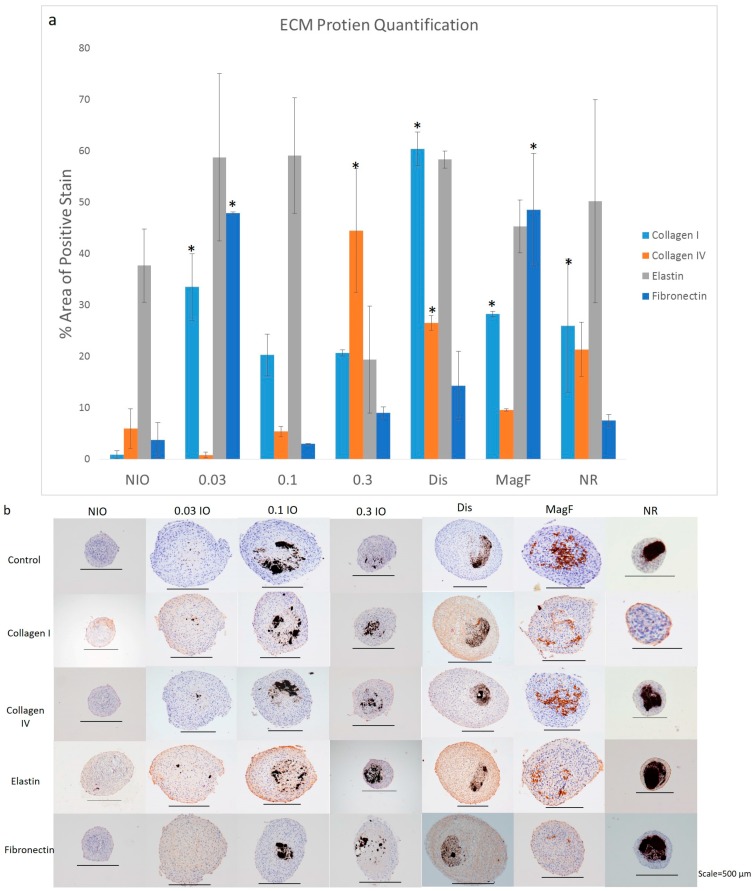
Immunohistochemistry Staining for MNP spheroids. Spheroids were fabricated with varying MNP concentrations, type, shape, incorporation method, or no MNP. (**a**) After day 40 time point, spheroids were collected, fixed and processed for immunohistochemical examination for collagen I, collagen IV, elastin and fibronectin. Image J was used to quantify the percent area of positive stain for the various samples. “*” represents samples that have ECM protein production that is significantly different from the NIO control (**b**) NIO, 0.03 mg/mL IO, 0.1 mg/mL IO, 0.3 mg/mL IO, dispersed (0.3 mg/mL IO), magnetoferritin, and nanorods stained for collagen I, collagen IV, elastin and fibronectin with a control that was not stained.

While the results from the previous section demonstrate an increase in collagen production, it is not clear if this increase is specific to one type of collagen or other ECM proteins. To determine the effect of MNP shape, type, level of concentration and incorporation method on ECM production, spheroids were immunohistochemically analyzed for four specific ECM proteins: collagen I, collagen IV, elastin and fibronectin. These ECM proteins were chosen because of their importance for cellular and tissue structure, organization and function [20]. Results demonstrated that each spheroid type stained positive for collagen I, collagen IV, elastin and fibronectin ECM proteins when compared to each of their negative controls, respectively (Figure 3b). Each spheroid type was also compared to the NIO control to determine the effect of MNP shape, type, level of concentration and incorporation method.

ImageJ was used to quantify the positive expression area of the ECM protein markers in Day 40 spheroids and normalized to the respective total area of the tissues using the method described by Vrekoussis et al. [30] (Figure 3a). The effect of varying the IO concentration on the ECM protein expression was first examined. As the concentration of IO was increased, the expression of collagen IV also increased. The highest concentration of IO (0.3 mg/mL) was significantly different from the NIO control with the highest percentage of the positive expression area at 44.5% compared to the control NIO with a percentage of positive expression area of 3.25%. However, the expression of collagen I decreased as the IO concentration was increased. Although only the lowest concentration of IO, 0.03 mg/mL IO was significantly different from the NIO control, there was an overall increase in the expression of both elastin and fibronectin compared to the control NIO. Nanorods were then used to determine how the shape of the MNP affected the stimulation of ECM proteins. Results showed an increase in all ECM protein production when compared to the NIO control. In comparison to spherical MNPs with an equivalent concentration, nanorods showed a greater stimulation of elastin with a positive expression area of 50.19%. Next, a comparison of the magnetoferritin to 0.3 mg/mL IO and the NIO control determined an increase in the expression of all of the ECM proteins. When compared to 0.3 mg/mL IO, magnetoferritin showed a higher area of expression for all ECM proteins, except for collagen IV, with fibronectin presenting the highest expression area of 56.28%. This area was also the highest expression of fibronectin from all of the MNPs in the study. Finally, the dispersed incorporation method results showed the highest expression for collagen I, with a total positive expression area of 72.47%. An increase in the positive expression of all the ECM proteins was also observed, unlike the NIO control.

**Figure 4:**
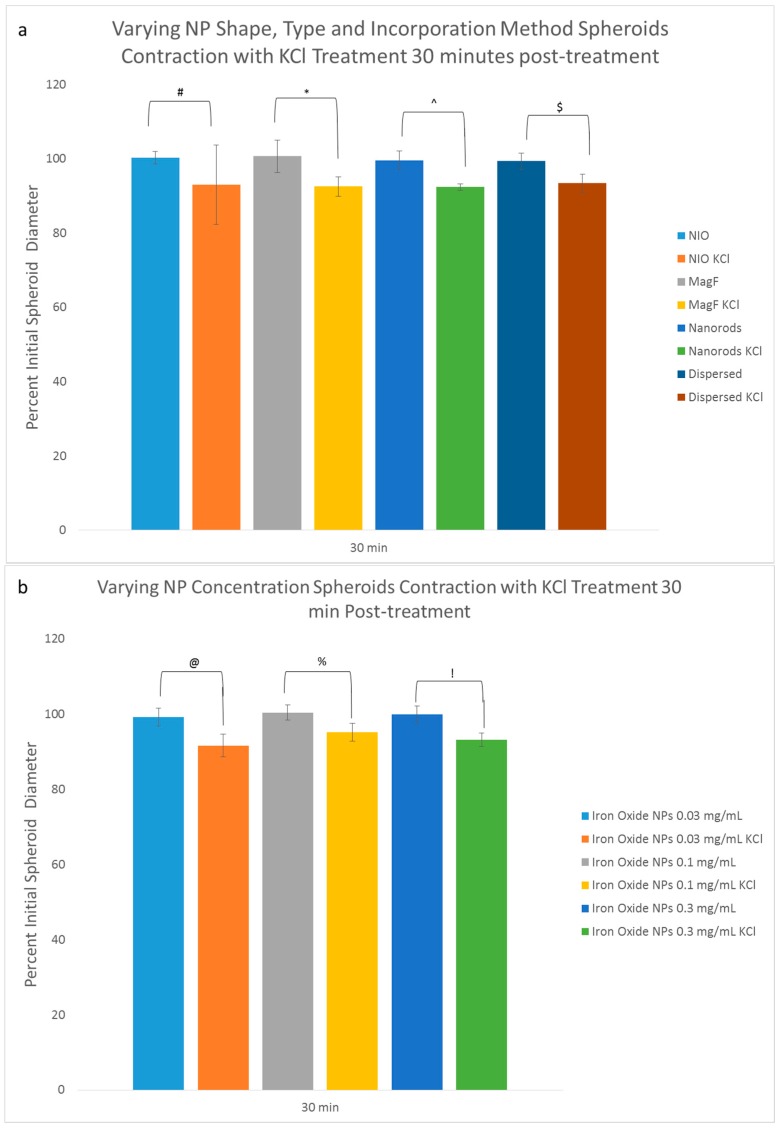

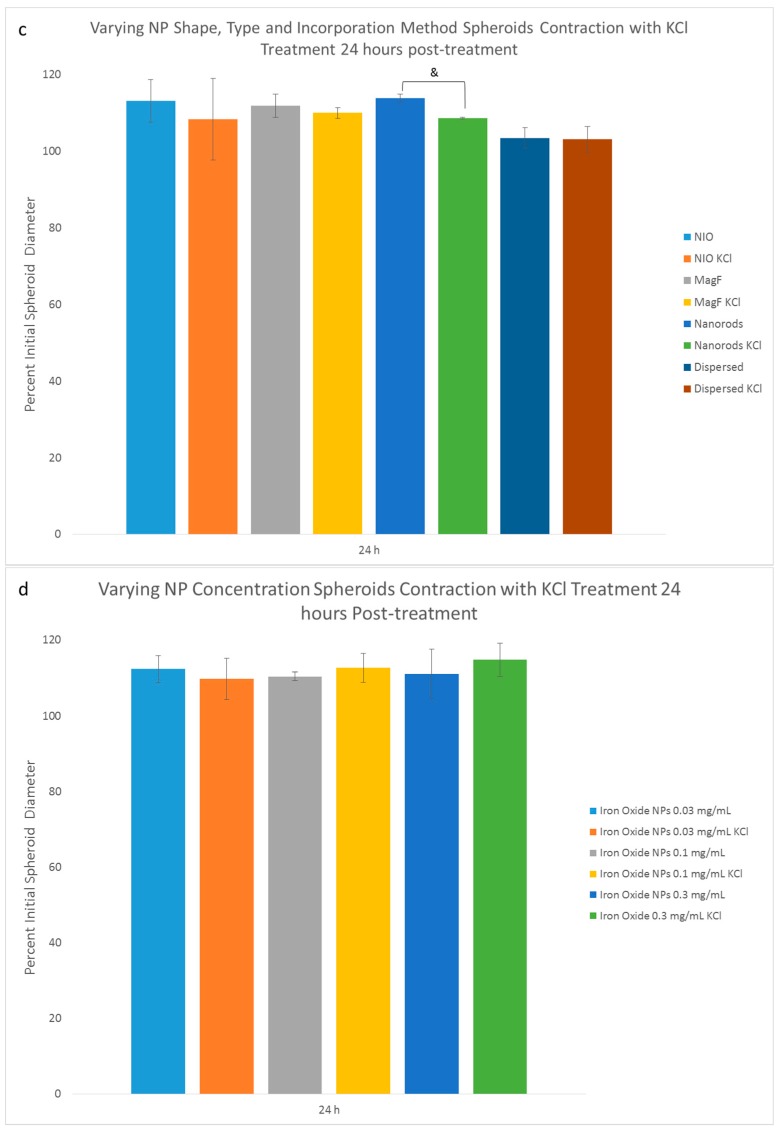

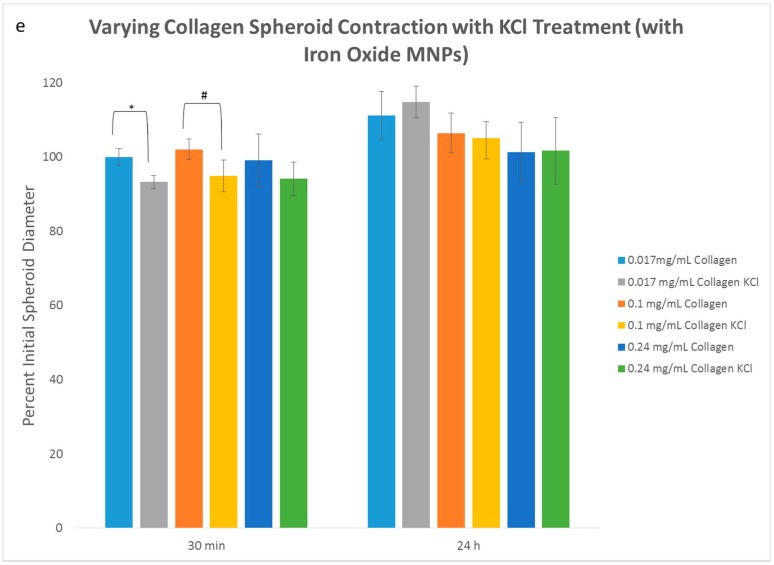
Potassium Chloride Functional Assay. (a–d) Spheroids were fabricated with varying NP concentrations shape, type, and incorporation method. Two sets of spheroids were collected from each group after 3 days of fusing (*n* = 5). One from each set were treated with KCl and the other set was the control. Spheroids were imaged before KCl treatment, 30 min and 24 h after KCl treatment. Percent initial spheroid diameter for KCl treated samples were compared to their counterparts without the treatment at 30 min and 24 h. The results indicate that there is statistical difference between all the treatment and non-treatment groups at 30 min and only nanorods at 24 h showed a significant difference in percent initial diameter between KCl treatment and non-treatment samples. This statistical difference is represented by “#”, “*”, “^” “$”, “@”, “%”, “!”, and “&” for (a–d). This suggests that none of the experimental groups have an adverse effect on the functionality of the spheroids; (**e**) Spheroids were fabricated with varying amounts of collagen content. Two sets of spheroids were collected from each group after 3 days of fusing (*n* = 5). The results indicate that there is a statistical difference (represented by “*” and “#”) after 30 min of KCl treatment between experimental group and control for 0.017 mg/mL and 0.1 mg/mL of collagen. After 24 h there is no statistical difference between the groups.

Since these results did validate the effect that MNPs have on ECM production it was important to next investigate their effect on cellular function. A subsequent analysis of the effect of MNPs on the contractile function of the cells determined the importance of potassium channels in the contraction and relaxation of smooth muscle cells (SMC) [31,32,33,34]. Potassium and sodium ions work in tandem to relax and contract the cell through positive and negative charges of the plasma membrane caused by concentrations of each ion within SMCs [35]. Alterations to potassium channels can affect the size, functionality, and tone of SMCs [32,34]. It was then necessary to utilize a functional assay to determine if MNPs had an effect on the function of SMCs because such a quantitative measure the specific functions of contraction could determine if the functional activity of the cells fell within a normal range. Results demonstrated a statistical difference between all the KCl treated and non-treated groups at 30 min; only the nanorods at 24 h showed a significant difference in the initial diameter (in terms of percentage) between the KCl treated and non-treated control samples. A significant difference in the percentage of the initial diameter of nanorod, dispersed, and 0.03 mg/mL IO spheroids was observed 30 min after treatment compared to their non-treated counterparts (Figure 4a,b). The only group to exhibit any significant difference in the percent initial diameter between treatment and non-treatment was nanorod spheroids, a difference which was observed 24 h after KCl treatment (Figure 4c,d).

This same experiment was performed with spheroids of varying collagen content and constant IO content (Figure 4e). The rationale was to determine how the concentration of collagen affects contraction and relaxation of spheroids when exposed to potassium chloride. Fusion was not deemed to be a factor in this assay because the test was performed on individual spheroids. Results demonstrated that an increase in the collagen content (0.24 mg/mL) resulted in less relaxation over time. At 30 min, a statistical difference was observed between the KCl treated MNP spheroids with 0.017 mg/mL and 0.1 mg/mL collagen content when compared to their respective controls that contained no exogenous collagen type I. However, after 24 h no statistical difference was observed between any of the groups and their controls.

For the final KCl experiment, mini tissues were used to examine the effect of MNP incorporation on the fusion process of spheroids to each other in addition to contraction and relaxation. The rationale was to determine how the incorporation of MNPs into spheroids affects the fusion of spheroids into mini tissues and the resulting ability of these mini-tissues to contract and relax. After 30 min of incubation with KCl, the media was removed and replaced with fresh media and all of the samples were imaged and diameters measured. The KCl treated mini tissues contracted to 92% of its initial diameter after 30 min while the control mini tissues were 101% of its initial diameter (Figure S1). At 72 h the KCl treated mini tissues measured 88% of their initial diameter and the control mini tissues measured 96% of their initial diameter. A statistical difference was observed in the size of the KCl treated mini-tissues when compared to control cultures.

## 4. Discussion

An optimally biomimetic tissue engineered construct will have a network of ECM that mimics the native tissue. Although the effects of MNPs on cell viability, phenotype and to an extent gene expression are known, how MNPs will affect ECM and the cellular function of contraction and relaxation is minimally understood [13,24]. Here, we investigated the influence of MNP concentration, type and method of incorporation on ECM production and cell contractility and relaxation.

The effect of the MNP concentration was first examined. Gardi et al. showed that changes in iron concentration can modulate collagen production [35]. In addition to stimulating matrix synthesis, iron also plays a role in matrix remodeling and degradation [29]. Our findings were supported by these studies in that they suggest that higher concentration of iron oxide stimulates greater collagen production, which is perhaps due an increase in the iron ions present from degradation of the nanoparticles over time that stimulates increased ECM production by SMCs [6]. Our findings were also supported by the studies of Briley-Saebo et al., in which they observed a degradation of superparamagnetic iron oxide nanoparticles within hepatic cells following a single injection intravenously in rats [36]. Furthermore, IHC results suggest that as the concentration of IO increased the production of collagen IV increased while the production of collagen I decreased. However, an examination of all of the IO concentrations with the exception of 0.3 mg/mL IO, determined that the relative amount of collagen I was higher than collagen IV. These results were mostly expected because collagen I, which is a fibril-forming collagen that provides structure and stability for tissues, is usually the most abundant collagen type [18]. However, the increase in collagen IV production is important because collagen IV is a network-forming collagen that is a major structural protein of basement membranes, thereby providing structural stability [18]. To our knowledge, this is the first such study elucidating the specific production of collagen IV compared to collagen I when cells are exposed to IO MNPs. This study is critical because IO MNPs can enhance structural stability of tissues, which is expected to affect tissue functions such as contraction and relaxation. Our IHC results also demonstrate that varying the concentration of IO has an effect on the production of elastin, which is supported by the work of Bunda et al. [21]. They demonstrated that, within a specific range of 2–20 μM of iron, there will be a positive effect on elastogenesis while concentrations outside of this range decrease elastin production [21]. Elastin is an important ECM component because it provides elasticity to the tissue and plays a role in cell adhesion, migration, and signaling [37]. Finally, our results for the cellular functions of contraction and relaxation showed that the concentration of IO did not have a significant effect, which demonstrates that a variance in the concentration of IO does not adversely affect the functionality of SMCs after 24 h.

We next examined the effect of MNP type on the production of ECM in spheroids by comparing magnetoferritin with the control of no MNPs. It was determined that magnetoferritin caused the greatest stimulation of fibronectin when compared to NIO control as well as the varying concentrations of IO. Within the ECM, the role of fibronectin involves mediating a variety of cellular interactions including cellular adhesion, migration, and growth [38]. The apoferritin shell of this MNP can influence the production of ECM because of the interaction of cells with ferritin, which is a form of stored iron found in the body [23]. It induces the oxidation of Fe (II) to Fe (III) thus facilitating the transfer of iron to ferritin. Ferritin is also necessary in iron homeostasis within the body in that it stores and releases this surplus of iron when as necessary [39]. Although iron overload can lead to ECM degradation, fibrosis and cell death from the overproduction of reactive oxygen species (ROS), we observed no adverse effect on the cellular function of contraction and relaxation after 24 h (Figure 4a–d) [23]. The collagen quantification of magnetoferritin supports this result in that no significant increase in collagen production was observed at day 3. However, the results obtained from our collagen quantification indicated a significant increase in collagen production at day 40, possibly indicating an effect on the contraction and relaxation function of the spheroid at a later time point. Although iron oxide could also have either a direct or indirect effect on cell functions such as migration, proliferation and differentiation, which was not investigated in this study [35].

Nanorod MNPs were used to examine the effect of MNP shape on ECM production and cellular contraction and relaxation. An equivalent mass of cylindrically shaped nanorods, 85 nm in diameter by 425 nm in length, was used for spheroid formulations, along with spherical nanoparticles, which had a diameter of 30 nm. In comparison to spherical nanoparticles, the nanorods demonstrated a significant increase in the specific stimulation of elastin and collagen IV. This stimulation was perhaps caused by the increased rate of internalization and further translocation of either the cylindrical or rod-shaped nanoparticles within the cells [15]. Such an increase could in turn increase the higher intracellular level of iron which may have an effect of the expression of genes that encode for ECM components. For example, in their study of genes regulating ECM production, Templeton and Liu suggested that this regulation was dependent on a base level of iron, a deviation from which would in turn affect the expression of these genes [24]. The dispersion method was next used to examine the effect of the method of MNP incorporation on ECM production. The increase in collagen production shown in IHC is perhaps due to the increased interaction between cells and iron from the dispersal of the iron oxide throughout the spheroid. The interaction of MNPs, a naturally stiff substrate, with cells in turn causes a redistribution of the cytoskeleton, a reinforcement of the linkages and changes in cell motility [40]. These changes in turn exhibit a mechanical stress on the cells that can result in an upregulation of the production of ECM proteins [40]. It is also possible that the modulation of collagen expression via iron ions may cause this increased production of collagen [29].

We determined that it was possible to stimulate SMCs to increase the production of collagen I and IV over other ECM proteins by varying the concentration of IO and by using different incorporation methods. Such a variance may be efficacious for use in skin treatment applications or for the treatment of injured or degenerated cartilage. Kim et al., for example, utilized gold nanoparticles to inhibit the formation of advanced glycation end products (AGEs) for anti-aging effect [25]. In our study, to stimulate specific ECM proteins using specific type of MNP, we determined that magnetoferritin favors the synthesis of fibronectin over collagen and elastin, which can translate into vascular implant applications via cellular adhesion. Similarly, Shimizu et al. utilized magnetic labeling of fibroblasts to improve cell seeding efficiency into 3D porous scaffolds for vascular applications [41]. It was determined that shape as well as the concentration of IO affected the stimulation of elastin, with nanorods and a 0.1 mg/mL of IO yielding the greatest stimulation of elastin. This concept can be applied for the engineering of elastic arteries used in cardiovascular tissues. However, the presence of iron MNPs poses concerns regarding the translation of tissue engineered constructs into a clinical setting. Previous work in the lab has shown that MNPs can be coated with biodegradable polymers to degrade up to 36% of the iron oxide in cellular spheroids over 21 days [6].

Although collagen is important for the structural stability of tissues, an abundance of ECM can interfere with cellular contraction and relaxation. An increased percentage of collagen present in the tissue can in turn decrease the resiliency of the tissue, however by altering the mechanical properties [42]. As stated earlier, our results demonstrated that IO nanoparticles can stimulate the overall production of collagen overtime. Therefore, it is necessary to determine to what affect any varying of the collagen content will have on the contraction and relaxation of cells. Determining this variation is important for selecting MNPs that can affect the tissue function. Results for this study demonstrated that high collagen contents inhibit contraction, which was expected, while low collagen contents allowed for contraction and relaxation. For the final cell functionality test with mini tissues the results demonstrated a significant difference over time in the size of tissues treated with KCl in comparison to their controls. This difference indicates that individual spheroids maintain cell function and that a fully fused developing tissue will also respond, thus indicating that the mechanism of cellular fusion does not affect overall cell function.

## 5. Conclusions

IO nanoparticles incorporated into cellular spheroids exhibited a demonstrable effect on ECM production, particularly on the stimulation of specific ECM proteins. An increase in the concentration of IO nanoparticles resulted in an increase in the production of collagen IV. Varying concentrations of IO also played a role in the specific stimulation of the ECM protein elastin. It was also determined that the biological MNP, magnetoferritin, increased the production of all the ECM proteins, particularly the protein fibronectin. Nanorods favored the stimulation of elastin, meaning the shape of the nanoparticle played a part in specific stimulation of ECM proteins. The incorporation method also had an effect on the specific stimulation of collagen over the other ECM proteins. Finally, no adverse functional effects of the MNPs were observed to hinder the ability of cells to contract. Future work will involve investigating the effects of varying NP on elastin content in cellular spheroids and the effect of varying NP on other cell types such as cardiac cells or aortic fibroblasts.

## Supplementary Materials

The following are available online at www.mdpi.com/2306-5354/4/1/4/s1, Figure S1: Potassium Chloride Assay on “mini” tissues. 25 spheroids with 0.3 mg/mL IO were fabricated and fused into mini tissue constructs. Results of the KCl functional assay indicate that there is a statistical difference in size over time of the tissues in comparison to the controls, Figure S2: TEM of (**a**) spherical nanoparticles and (**b**) nanorods at 100,000× magnification.
