# Supplementary material for: Iron Oxide Nanoparticles Stimulates Extra-Cellular Matrix Production in Cellular Spheroids
Source: Bioengineering (Basel). 2017 Jan 21;4(1):4. doi: 10.3390/bioengineering4010004 (PMC5590449; doi:10.3390/bioengineering4010004)
Supplement: Supplementary file 1 [file bioengineering-04-00004-s001.pdf]

Supplementary Materials: Iron Oxide Nanoparticles Stimulates Extra-Cellular Matrix Production in Cellular Spheroids

Megan Casco, Timothy Olsen, Austin Herbst, Grace Evans, Taylor Rothermel, Lauren Pruett, Dan Simionescu, Richard Visconti and Frank Alexis

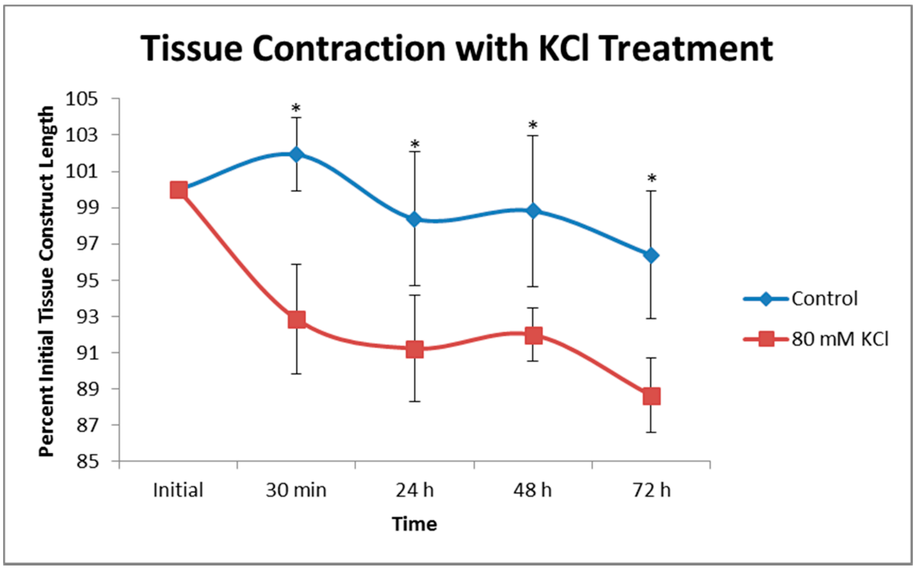

Figure S1. Potassium Chloride Assay on "mini" tissues. 25 spheroids with 0.3 mg/mL iron oxide (IO) were fabricated and fused into mini tissue constructs. Results of the KCl functional assay indicate that there is a statistical difference, represented by '*', in size over time of the tissues in comparison to the controls.

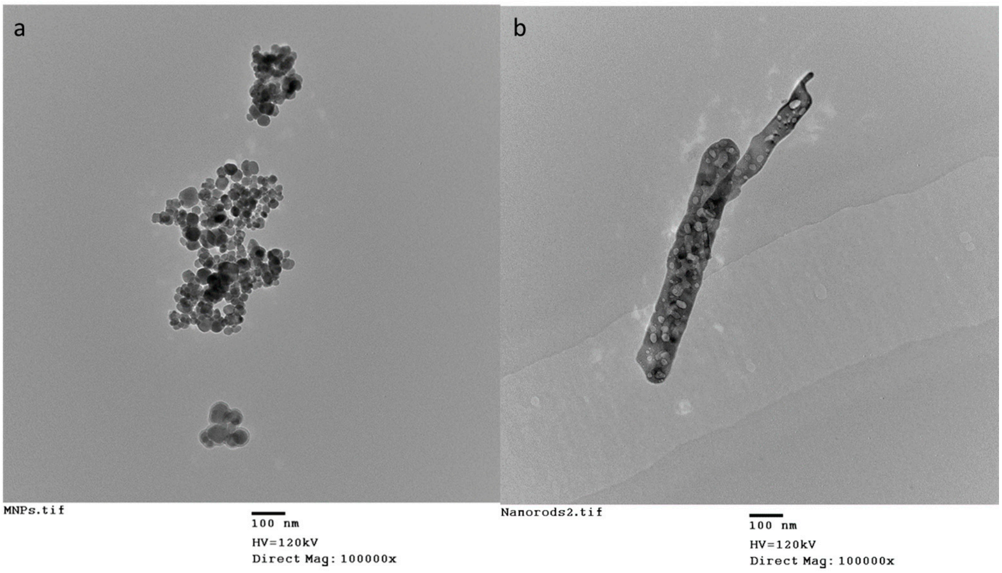

Figure S2. TEM of (**a**) spherical nanoparticles and (**b**) nanorods at 100,000× magnification.

© 2017 by the authors; licensee MDPI, Basel, Switzerland. This article is an open access article distributed under the terms and conditions of the Creative Commons Attribution (CC BY) license (http://creativecommons.org/licenses/by/4.0/).
